# Feasibility of Using a Commercial Board Game to Assess Upper Extremity Function in Older Adults

**DOI:** 10.1093/geroni/igab046.3699

**Published:** 2021-12-17

**Authors:** Allison Niemiec, Yareli Lopez Hernandez, Ejlal Bin Mulayh, Rachel Logue, Susan Brown

**Affiliations:** 1 University of Michigan, Ann Arbor, Michigan, United States; 2 University of Michigan, University of Michigan, Michigan, United States

## Abstract

Upper extremity function, particularly the hand, declines with aging and is predictive of executive ability and independence. Standard assessments typically focus on strength partly due to a lack of easily administered functional tasks requiring multi-joint coordination and precision grasp. This study aimed to determine the feasibility of using an inexpensive board game to assess upper extremity function in older adults. Six healthy older adults (77 +/- 5.1 years) completed reaching tasks using the Connect4® game that requires grasping and placing small discs into a vertical board. Tasks included different hand configurations (unilateral, bilateral), and two dual-task conditions (serial subtraction by 7s and placing colored discs to match specific color patterns). The time to complete each task was recorded. For comparison purposes, participants completed a standardized pegboard test (Purdue Pegboard) using one or both hands. Connect4 results were similar to age-normative findings reported for the Purdue Pegboard. Dominant versus non-dominant hand performance did not differ while bilateral coordination tasks were slower than unilateral tasks for both the Purdue Pegboard (p<0.05) and Connect4 (p<0.01). Pegboard and Connect4 times were moderately to strongly correlated for all hand configurations. Dual-task conditions using Connect4 led to longer completion times (p<0.05). Preliminary results support the use of Connect4 as a functional upper extremity assessment tool for older adults. It is inexpensive, engaging, easy to use, and allows for cognitive-motor assessment using dual-task protocols, a critical factor in maintaining functional independence in older individuals. Further research will include a formal validation study across a wider age range.

